# A comprehensive assessment of personality traits and psychosocial functioning in parents with bipolar disorder and their intimate partners

**DOI:** 10.1186/s40345-019-0172-x

**Published:** 2020-02-10

**Authors:** Lisa Serravalle, Vanessa Iacono, Sheilagh Hodgins, Mark A. Ellenbogen

**Affiliations:** 1 Centre for Research in Human Development, Concordia University, Montréal, Canada; 2 Département de Psychiatrie, Université de Montréal, Montréal, Canada; 3 Department of Clinical Neuroscience, Karolinska Institute, Stockholm, Sweden

**Keywords:** Bipolar disorder, Intimate partners, Personality, Psychosocial functioning, Marital adjustment

## Abstract

**Background:**

Individuals with bipolar disorder (BD) often possess maladaptive traits and present with various difficulties in psychosocial functioning. However, little is known about the intimate partners of adults with bipolar disorder (BD) and how mental illnesses other than BD within couples may further complicate the picture. Such knowledge is needed to inform both couple and family interventions.

**Methods:**

Participants were parents whose children were enrolled in a prospective study: 55 with BD and their partners, and 47 healthy control couples. All completed diagnostic interviews, and questionnaires describing personality traits, negative life events, coping skills, social support, marital adjustment and inter-partner verbal aggression. Parents with BD and healthy control parents were compared, as were the intimate partners. A series of exploratory analyses focused on the average measures within couples, with and without BD, and took account of comorbid personality disorders among those with BD and major depressive disorder among their partners.

**Results:**

Intimate partners of adults with BD, relative to healthy control partners, presented with more mental disorders, higher neuroticism, lower extraversion, more emotion-focused coping, smaller social networks, less satisfaction with their social networks, and little, satisfying social contact. Additionally, they reported less consensus and satisfaction in their marital relationships, and engaged in more verbal aggression towards their partners. Participants with BD showed similar, more extreme, characteristics. Marital distress and verbal aggression were greatest among couples with an adult having BD and a comorbid personality disorder or a partner with major depressive disorder.

**Conclusion:**

This study contributes to the literature by demonstrating that *both* parents with BD and their intimate partners exhibit high levels of mental illness, maladaptive personality traits and psychosocial difficulties, thus limiting their partners’ ability to provide support and stability in the these high risk families. Moreover, mental illnesses other than BD may contribute to marital problems within couples. Some statistical analyses, particularly those involving comorbid conditions, were under-powered in this study. As clinical implications, the current study suggests that *both* individuals with BD and their partners could benefit from interventions aimed at lowering emotionality and verbal aggression, and increasing social support and effective coping skills.

## Background

Bipolar disorder (BD), a chronic and debilitating condition, is ranked among the top ten leading causes of disability worldwide (World Health Organization 2001). In addition to the incapacitating effects of acute symptoms, persons with BD display maladaptive personality traits and impaired psychosocial functioning between episodes (Hodgins et al. 2002). Adults with BD also experience high levels of dependent negative life events (Bender et al. 2010; Ellenbogen and Hodgins 2004), and engage in ineffective coping strategies to address stressful situations (Fletcher et al. 2013; Moon et al. 2014). Additionally, there is evidence to suggest individuals with BD have difficulty establishing and maintaining social support networks that could aid in buffering stress (Beyer et al. 2003; Eidelman et al. 2012). Further complicating the picture, individuals with BD present with high rates of personality disorders (PDs; Brieger et al. 2003; Fan and Hassell 2008; George et al. 2003). Among adults with BD, those with comorbid disorders, relative to those without, show greater impairment in interpersonal functioning (Carpenter et al. 1995; Loftus and Jaeger 2006). Taken together, individuals with BD present with stable maladaptive traits and experience recurring stress which they are unable to effectively cope with and further compounded by low levels of social support. Targeting these factors with effective treatments could potentially improve the course of BD and ameliorate the child-rearing environment. To inform such interventions, more information is needed about these factors within the family context.

Many adults with BD form intimate relationships that they report to be unsatisfactory (Whisman 2007) and characterized by verbal aggression from their partner (Lam et al. 2005). Moreover, divorce rates are two to three times higher in adults with BD relative to the general population (Kogan et al. 2004; Suppes et al. 2001). One factor contributing to marital instability in these couples may be the presence of a major affective disorder in the partner, which is approximately three-to-four times greater in partners of adults with, than without, BD (Butterworth and Rodgers 2008; Mathews and Reus 2001; Nordsletten et al. 2016). Little else is known about the characteristics of the intimate partners selected by adults with BD. There is some evidence to suggest that partners of individuals having BD may also display dysfunctional psychosocial patterns, such as adopting ineffective coping styles (Borowiecka-Karpiuk et al. 2014). Therefore, the intimate partner’s personality traits, dependent negative life events, coping skills and social support could potentially lessen or exacerbate maladaptive behaviours of their spouse, and be associated with marital adjustment and family functioning.

The intimate partners of adults with BD play key roles within the family context, particularly in rearing children. Relative to the children of families with parents having no mental disorders, the offspring in families with a parent having BD are at increased risk of developing internalizing and externalizing problems in childhood, interpersonal difficulties, risky sexual behaviors, low occupational competence, and mental disorders (Bella et al. 2011; Duffy et al. 2014; Nijjar et al. 2014; Ostiguy et al. 2009,2012; Shaw et al. 2005). In addition to genetic effects (Kieseppä et al. 2004; Song et al. 2015), parents with BD and their partners may contribute to poor functioning and increased risk for mental disorders among their offspring by adopting suboptimal parenting practices and maladaptive behaviors, often associated with high neuroticism, that contribute to a chaotic home environment (Ellenbogen and Hodgins 2004; Iacono et al. 2018). However, intimate partners of parents with BD might mitigate some of these negative effects in the home. For example, intimate partners could help individuals with BD identify signs of developing episodes, provide support for taking medication, and encourage participation in programs aimed at reducing maladaptive behaviors, and increasing effective coping skills and social support. Indeed psychosocial interventions, including those which include family members in the treatment of BD, have been shown to improve outcomes (Miklowitz 2006; Rea et al. 2003). Therefore, intimate partners may also be key participants in family-based interventions aimed at promoting healthy development of the offspring of parents with BD (OBD). To date, there are few empirical studies of intimate partners of individuals with BD. Thus, gaining knowledge of the psychosocial functioning and maladaptive traits of the intimate partners of parents with BD is needed.

The present study examined the mental health, personality traits, negative life events, coping skills, social support, marital adjustment and verbal aggression of adults with and without BD and their intimate partners. Participants were the parents and their spouses or intimate partners who participated in a prospective longitudinal study of families in the province of Quebec (Canada) having a parent with BD or parents with no mental disorder. The study included a comprehensive assessment of families (Ellenbogen and Hodgins 2004; Rende et al. 2005), their relatives (via a structured family history interview) and their offspring who were between 4 and 14 years of age, followed by a second assessment of the offspring approximately 11 years later (Nijjar et al. 2014; Ostiguy et al. 2012). The present study utilizes data collected in parents during the initial assessment and is different from a previous publication that focused on the effects of parents’ levels of neuroticism on measure of the family environment and their offspring (Ellenbogen and Hodgins 2004). As the first goal, to confirm and extend past findings, parents with BD were compared to healthy control parents. To meaningfully characterize the intimate partners of adults with BD, they were compared to partners of healthy control adults. In a second set of analyses, we focused on couples, to assess family-wide risk, comparing those with one partner with BD and those with two healthy control partners. Since comorbid PDs are common among adults with BD (Brieger et al. 2003; Fan and Hassell 2008; George et al. 2003), we conducted exploratory analyses to determine if couples that included a partner with BD and a PD differed from those without the comorbid disorder. Finally, given the elevated rate of major depressive disorder (MDD) among partners of adults with BD (Butterworth and Rodgers 2008; Mathews and Reus 2001; Nordsletten et al. 2016), we compared couples in which one partner presented BD with and without a partner with MDD to healthy couples.

## Methods

### Participants

Fifty-eight parents with BD and their 62 intimate partners from were recruited from psychiatric outpatient clinics and support groups in the province of Québec (Canada). Using community advertisements, healthy control parents (52 index parents and 48 spouses/intimate partners), who were free of any *current* Axis-I disorder or a history of affective disorder, were recruited from the same geographic regions as parents with BD. Six parents from families having a parent with BD and 10 parents from the control group were excluded from the study or dropped out prior to completing the baseline assessment. Thus, the final study sample included 102 index parents (55 BD and 47 control) and 102 intimate partners (59 BD and 43 control) from 204 parents participating in a prospective study comparing the development of children of parents with BD and children of healthy control parents (Ellenbogen and Hodgins 2004; Ostiguy et al. 2012). The majority of intimate partners were biological parents of the OBD with the exception of eight stepparents (4 males) in the families with a parent having BD. Eleven families consisted of single-parent families (7 BD, 2 males). Among the control families, one parent was randomly designated as an index parent. At the time of the assessments, index parents with BD, their intimate partners, index control parents, and their intimate partners had a mean age of 39.25 (*SD* = 5.35), 39.41 (*SD* = 5.06), 38.15 (*SD* = 5.24) and 38.28 (*SD* = 4.18) years respectively. Parent education level (number of years), used as a proxy of socioeconomic status, was 13.87 (*SD* = 2.96), 13.86 (*SD* = 3.39), 15.98 (*SD* = 2.88) and 15.35 (*SD* = 2.65) for index parents with BD, their intimate partners, index control parents, and their intimate partners respectively.

Diagnoses were confirmed using a semi-structured diagnostic assessment (see below) and psychiatric records. Some healthy control parents did meet criteria for *past* mental illness and personality disorders: 6 (13%) drug abuse/dependence, 2 (4%) anxiety disorders, 1 (2%) avoidant PD, 1 (2%) obsessive–compulsive PD and 1 (2%) PD NOS. For inclusion, all parents were required to have at least one biological child between 4 to 14 years of age, be fluent in English or French, and have been raised and educated in Canada. Parents who presented with a chronic medical condition, physical handicap, or below-average intelligence quotient (IQ < 70) were excluded. Parents were mostly Caucasian, middle-class, and French Canadian.

## Measures

### Diagnostic interviews

#### The structured clinical interview for DSM-III-R (SCID-I; Spitzer et al. 1992)

The SCID-I, a valid and reliable diagnostic instrument, was used to assess parents’ mental health (e.g., Zanarini and Frankenburg 2001). Independent inter-rater agreements were obtained on 15% of the interviews. Agreement between clinicians was excellent as indicated by the kappa coefficients for diagnoses of bipolar disorder, 1.0, and other mood disorders 1.0, (lifetime and current).

#### The structured clinical interview for DSM-IV axis II personality disorders (SCID-II; Gibbon et al. 1997)

The SCID-II was also administered to parents to assess the presence of personality disorders.

### Questionnaires

#### NEO personality inventory-revised (NEO PI-R; Costa and McCrae 1992)

The NEO PI-R is a self-report personality inventory. It includes 240 items measuring levels of trait neuroticism, extraversion, agreeableness, openness to experience, and conscientiousness using a Likert scale ranging from 1 (*strongly disagree*) to 5 (*strongly agree*). Studies have demonstrated high internal consistency (Chronbach’s α = .89 to .95), convergent and discriminant validity, as well as temporal stability of the NEO PI-R (Costa et al. 2000; Costa and McCrae 1992). Similar psychometric proprieties have been reported for its French translation (Rolland et al. 1998).

#### Dyadic Adjustment Scale (DAS; Spanier 1976)

The DAS is a 32-item, self-report questionnaire which evaluates overall relationship quality over the previous year within couples. The four subscales include consensus (agreement on matters important to the relationship), affectional expression (expression of affection and sexual desire), satisfaction (satisfaction with the relationship and commitment to its continuance), and cohesion (common interests and activities). Participants respond to each item using a Likert scale, with higher values indicating greater relationship quality. The DAS has adequate internal consistency (Chronbach’s α = .70 to .95). The DAS has been validated for its use in both English and French (Bouchard et al. 1991; Spanier and Thompson 1982). The DAS was only administered to parents with a current intimate partner.

#### Revised conflict tactics scales (CTS2; Straus et al. 1996)

The CTS2 measures self-reported levels of verbal and physical aggression within couples. Parents were asked to identify how often each of the 78 items occurred during the previous year*.* Adequate internal consistency, construct validity, and test–retest reliability have been demonstrated for the CTS2 (Straus and Mickey 2012; Vega and O’Leary 2007). Given the low base rate of physical violence, only levels of verbal aggression were utilized. The CTS2 was administered only to parents with a current intimate partner.

#### Arizona social support interview schedule (ASSIS; Barrera 1980; Barrera et al. 1981)

The ASSIS is a semi-structured interview containing 30 questions pertaining to the structural components of a participant’s social network (size and frequency of contact) and the adequacy of social support (satisfaction). Internal consistency (Chronbach’s α = 0.74 − 0.78) for the ASSIS are adequate (Barrera 1980; Barrera et al. 1981).

#### Psychiatric epidemiological research interview—Life Events Scale (PERI Life Events Scale; Dohrenwend et al. 1978)

The PERI Life Events Scale measures participants’ self-reported experiences of positive and negative life events which are coded as being dependent (e.g. divorce) or independent (e.g. a death in the family) of the participant’s own behaviour. Only negative dependent and independent life events were utilized due to their associations with mental illness (Kendler et al. 2010; Risch et al. 2009).

#### Coping inventory for stressful situation—adult (CISS; Endler and Parker 1994)

Parents rated the extent to which they engaged in 48 different coping activities following stressful situations using a five-point scale ranging from 1 (*Not at all*) to 5 (*Very Much*). Standardized *T* scores for three primary styles of coping (task-oriented, emotion-focused, and avoidance-oriented) were obtained. High internal consistency (Chronbach’s α = .78 − .88) and temporal stability have been reported for the CISS (Brands et al. 2014).

### Procedure

Data for the current study was collected from October 1994 to May 1997. Following a telephone screening, parents with BD were administered the SCID and SCID-II interviews in the laboratory or at their homes, as well as the NEO PI-R, CISS, ASSIS, DAS, and CTS2. Parents with BD were euthymic during testing. The intimate partners of parents with BD also completed the same interviews and questionnaires independently. The same procedure was undergone for healthy controls. Subsamples of parents and their intimate partners were contacted at a later date to complete the PERI-Life Event Scale. Informed written consent was obtained from all parents and procedures were approved by the Ethics Committee of the Université de Montréal (Montréal, Canada).

### Data analysis

Data were screened and corrected for outliers (the values of outliers was reduced or increased to three standard deviations from the mean) and distributional anomalies that violated statistical assumptions. A series of Multivariate Analysis of Covariance (MANCOVA) tests were conducted to examine differences in personality traits, negative life events, coping skills, social support, marital adjustment and verbal aggression between BD and healthy control index parents, and the partners of parents with BD and healthy control partners. MANCOVAs were also conducted to explore differences between couples with a parent having BD and a comorbid PD, couples having a parent with BD and no comorbid PD and healthy control couples. Similar analyses were conducted to examine the effects of having an intimate partner with a history of MDD in couples with a parent having BD. Due to the issue of non-independence and to obtain a global estimate of couple functioning, the latter analyses used mean scores across all available parents when there was more than one parent in a family. For the measure of verbal aggression at the couple level, we averaged the mean levels of verbal aggression towards and from partners (i.e., inter-partner verbal aggression). Education, a proxy for socio-economic status, was used as a covariate for all analyses. For all analyses, we focus on contrast analyses comparing groups across dependent variables of interest rather than multivariate omnibus tests, as the latter was not of interest in the present study (Kline 2008). From the effect size (Cohen’s d = 0.51) observed in a previous study of this population (Iacono et al. 2018), we estimated that the effect size was in the medium range (f^2^ = .15, R^2^ increment of .06 to .07). Therefore, a sample size of 92 was estimated to be sufficient to detect group differences in the aforementioned MANOVA analyses with power 0.80 and an alpha level of 0.05.

## Results

### Personality traits, negative life events, coping skills, social support, marital adjustment and inter-partner verbal aggression

#### Index parents with BD and healthy control partners

Of the index parents with BD, 15 (27%) also presented with PDs. As presented in Table 1, index parents with BD differed from index healthy controls on the majority of measures. Parents with BD, relative to index healthy controls, obtained higher scores for neuroticism, as well as lower scores for agreeableness and for conscientiousness. The effect size for neuroticism was particularly large, as group membership accounted for 25% of the residual variance. Relative to healthy index controls, parents with BD reported more dependent negative life events (*η*^2^ = .15). Parents with BD also reported less effective coping strategies compared to index healthy controls. In particular, 10 and 13% of the residual variance in emotion-focused and avoidance-oriented coping, respectively, was explained by group membership. Index parents with BD reported low social support, including smaller networks, less satisfaction, and fewer contacts, relative to index healthy controls. Group differences were largest (15% of the residual variance) for satisfaction with social network. Index parents with BD displayed less affection to their partner, were less satisfied with their relationship and perceived it as less cohesive, than index healthy control parents. Group membership explained between 6–8% of the residual variance in each of these areas of marital adjustment. Finally, parents with BD reported elevated levels of verbal aggression from their partner relative to healthy controls. `

**Table 1:** Comparisons of personality traits, psychosocial functioning, and marital adjustment of index parents with bipolar disorder (BD) and index healthy control parents and of intimate partners of parents with BD and intimate partners of healthy control parents

	Index parents				Intimate partners			
	BD	Healthy control			Of adults with BD	Of healthy controls		
	*M* (SD)	*M* (SD)	*F*	*η*^2^	*M* (SD)	*M* (SD)	*F*	*η*^2^
Personality (NEO-PI-R)^a^	*n* = 55	*n* = 47			*n* = 59	*n* = 43		
Neuroticism	60.40 (12.70)	45.11 (8.80)	33.41**	.25	50.49 (10.20)	44.58 (8.70)	7.41**	.07
Extraversion	48.47 (9.11)	51.45 (7.35)	1.62	.02	47.10 (7.87)	52.67 (7.22)	11.08**	.10
Openness	50.95 (10.50)	52.85 (8.65)	.01	.00	48.20 (8.35)	50.98 (7.04)	.99	.01
Agreeableness	49.56 (9.74)	54.04 (6.74)	4.08*	.04	51.19 (8.41)	49.67 (8.77)	1.14	.01
Conscientiousness	44.44 (11.10)	50.43 (6.07)	6.25*	.06	50.18 (9.84)	51.42 (7.74)	.19	.00

Negative life events (LES)^b^	*n* = 48	*n* = 32			*n* = 28	*n* = 33		
Dependent	2.83 (1.98)	1.09 (1.28)	13.41**	.15	1.93 (1.33)	1.58 (1.56)	.90	.02
Independent	.44 (.62)	.31 (.59)	.89	.01	.32 (.55)	.39 (.55)	.25	.00

Coping skills (CISS)^c^	*n* = 55	*n* = 47			*n* = 58	*n* = 43		
Task-oriented	44.80 (10.80)	51.60 (9.75)	5.82*	.06	49.40 (8.85)	51.88 (8.11)	.98	.01
Emotion-focused	55.27 (10.40)	46.70 (8.68)	11.39**	.10	49.90 (8.79)	45.63 (7.51)	5.69*	.06
Avoidance-oriented	53.04 (11.90)	44.62 (9.24)	15.10**	.13	48.34 (9.63)	46.37 (10.6)	1.07	.01

Social support (ASSI)^d^	*n* = 55	*n* = 47			*n* = 59	*n* = 43		
Size of social network	10.04 (6.66)	17.85 (13.80)	7.65**	.07	10.25 (7.74)	16.60 (12.40)	8.66**	.08
Satisfaction with social network	25.53 (3.56)	28.30 (2.01)	17.78**	.15	25.98 (3.46)	28.23 (2.45)	11.80**	.11
Amount of social contact	6.27 (6.98)	12.47 (11.8)	7.08**	.07	7.61 (7.91)	12.65 (11.8)	5.47*	.05
Satisfaction with social contact	4.25 (.95)	4.74 (.61)	6.40*	.06	4.14 (.97)	4.63 (.691)	7.09**	.07

Marital adjustment (DAS)^e^	*n* = 38	*n* = 45			*n* = 43	*n* = 40		
Consensus	50.87 (7.80)	53.62 (5.66)	2.46	.03	49.77 (8.12)	52.88 (6.54)	4.89*	.06
Affectional expression	8.24 (2.50)	9.31 (1.81)	7.05**	.08	8.00 (2.66)	9.10 (2.41)	3.47	.04
Satisfaction	35.71 (7.70)	39.56 (4.46)	5.54*	.07	32.63 (8.90)	39.53 (4.83)	17.49**	.18
Cohesion	13.79 (5.48)	16.18 (3.51)	4.94*	.06	13.81 (5.49)	15.68 (4.04)	3.60	.04

Verbal aggression (CTS)^f^	*n* = 38	*n* = 45			*n* = 44	*n* = 41		
From partner	18.39 (21.60)	8.62 (11.60)	3.97*	.05	14.98 (21.00)	10.61 (10.8)	.41	.01
Towards partner	16.71 (21.90)	8.73 (9.78)	3.76	.05	23.73 (26.80)	8.95 (9.31)	8.11**	.09

T-scores were used for the Personality and Coping Skills subcategories, *p < .05, **p < .01

BD: bipolar disorder

^a^From the NEO Personality Inventory-Revised

^b^From the Life Events Scale

^c^From the Coping Inventory of Stressful Situations

^d^From the Arizona Social Support Interview

^e^From the Dyadic Adjustment Scale

^f^From the Conflict Tactic Scale

#### Partners of index parents with BD and healthy control partners

The intimate partners of parents with BD and healthy control partners also differed. Among the intimate partners of parents with BD, 18 (31%) presented past or current major depression, 9 (15%) past or current alcohol abuse/dependence, 6 (10%) past or current drug abuse/dependence, 6 (10%) past or current anxiety disorders, 5 (8%) past or present eating disorders, 1 (2%) borderline PD, 1 (2%) avoidant PD, 1 (2%) narcissistic PD, 1 (2%) schizotypal PD, and 2 (3%) passive aggressive PD. As presented in Table 1, partners of index parents with BD, relative to healthy control partners, obtained higher scores for neuroticism and lower scores for extraversion, accounting for 10 and 7% of the residual variance respectively. Partners of parents with BD engaged in more emotion-focused coping relative to partners of healthy control index parents, with group membership explaining 5% of the residual variance.

Intimate partners of parents with BD also reported smaller social networks, less satisfaction with their social networks, and less satisfying social contact than the partners of healthy control parents. Similar to parents with BD, group differences were largest (11% of the residual variance) for satisfaction with social network. Additionally, they reported less consensus (6% of the residual variance) and satisfaction (18% of the residual variance) in their marital relationships, and engaged in more verbal aggression towards their partners (9% of the residual variance). Low couple satisfaction reported by the intimate partners of parents with BD, given the large effect size, was particularly robust.

In sum, index parents with BD differed from healthy control index parents as to high levels of maladaptive personality traits, dependent negative life events, ineffective coping skills, low levels of social support, unsatisfying marriages, and verbal abuse from their spouse. Their intimate partners differed from the healthy control partners to a lesser extent, but significantly as to the same personality traits, the use of emotion-focused coping skills, low levels of social support, as well as unsatisfactory marital relationships and verbally abusing their spouse.

#### Exploratory analyses comparing personality traits, negative life events, coping skills, social support, and marital adjustment and inter-partner abuse of couples with and without a partner with BD

The next series of analyses compared mean scores of couples with and without a partner with BD. As presented in Table 2, couples with one partner with BD, as compared to healthy control couples, obtained higher scores for neuroticism, lower scores for extraversion and conscientiousness. The couples with a partner with BD reported more dependent negative life events and more ineffective coping skills, less social support, poorer marital adjustment and more verbal aggression than healthy control couples .

**Table 2:** Comparisons of personality traits, psychosocial functioning and marital adjustment of couples with and without a partner with bipolar disorder

	Couples			
	One partner with BD	Two healthy control partners		
	*M* (SD)	*M* (SD)	*F*	*η*^2^
Personality (NEO-PI-R)^a^	*n* = 58	*n* = 47		
Neuroticism	55.37 (7.43)	44.94 (7.35)	35.86**	.26
Extraversion	47.88 (6.45)	52.10 (5.56)	7.80**	.07
Openness	49.68 (7.70)	52.10 (6.46)	.13	.00
Agreeableness	50.11 (6.81)	52.16 (5.71)	.51	.01
Conscientiousness	47.12 (7.73)	51.06 (5.45)	6.21*	.06

Negative life events (LES)^b^	*n* = 48	*n* = 33		
Dependent	2.55 (1.47)	1.32 (1.17)	13.17**	.14
Independent	.40 (.48)	.35 (.40)	.61	.01

Coping skills (CISS)^c^	*n* = 57	*n* = 47		
Task-oriented	46.93 (7.43)	51.60 (7.20)	5.51*	.05
Emotion-focused	52.56 (7.13)	46.19 (6.08)	13.41**	.12
Avoidance-oriented	51.33 (8.15)	45.38 (7.60)	14.20**	.12

Social support (ASSI)^d^	*n* = 58	*n* = 47		
Size of social network	10.16 (5.72)	17.20 (9.22)	12.52**	.11
Satisfaction with social network	25.75 (2.89)	28.27 (1.85)	21.07**	.17
Amount of social contact	7.00 (6.02)	12.45 (8.10)	8.73**	.08
Satisfaction with social contact	4.19 (.76)	4.70 (.50)	10.33**	.09

Marital adjustment (DAS)^e^	*n* = 45	*n* = 46		
Consensus	50.39 (6.72)	53.52 (5.44)	6.51*	.07
Affectional expression	8.08 (2.41)	9.23 (1.95)	6.65*	.07
Satisfaction	34.07 (7.80)	39.72 (4.22)	15.83**	.15
Cohesion	14.04 (4.89)	16.15 (3.32)	5.25*	.06

Verbal aggression (CTS)^f^	*n* = 45	*n* = 45		
Inter-partner	18.45 (19.47)	9.44 (9.34)	3.46	.04

Due to non-independence of data and to obtain a global estimate of couple functioning, mean scores across all available parents. when there was more than one parent in a family were used (number of single-parent families = 11 (7 BD))

T-scores were used for the Personality and Coping Skills subcategories, *p < .05, **p < .01

BD: bipolar disorder

^a^From the NEO Personality Inventory-Revised

^b^From the Life Events Scale

^c^From the Coping Inventory of Stressful Situations

^d^From the Arizona Social Support Interview

^e^From the Dyadic Adjustment Scale

^f^From the Conflict Tactic Scale

#### Couples in which one partner presents BD and a PD

As presented in Table 3, couples that included a partner with BD and a PD as compared to those with BD and no PD, obtained lower scores for avoidant coping (22% of the variance), and reported less consensus (12% of the variance) and satisfaction (20% of the variance) with their marital relationship. Notably, the proportion of residual variance explained by group membership was 1.3 to 2 times greater than in the analyses which compared *all* couples with a parent having BD vs. controls (see Table 2). Increased inter-partner violence was found in couples with a parent having BD and a PD relative to healthy control couples; this difference was not found between couples with a BD parent and no PD and control couples. Approximately 5% of the residual variance in inter-partner violence was explained by group membership. On most measures, however, both types of couples with a partner with BD differed from healthy couples.

**Table 3:** Comparisons of personality traits, psychosocial functioning and marital adjustment of couples with a partner with bipolar disorder, with and without a comorbid personality disorder, and healthy control couples

	Couples					Couples with a partner with BD and PD vs healthy control couples	Couples with a partner with BD no PD vs healthy control couples	Couples with a partner with BD and PD vs couples with a partner with BD no PD
	One partner with BD		Two healthy control partners					
	PD	No PD						
	*M* (SD)	*M* (SD)	*M* (SD)	*F*	*η*^2^			
Personality (NEO-PI-R)^a^	*n* = 21	*n*= 37	*n*= 47					
Neuroticism	58.52 (7.40)	53.59 (6.93)	44.94 (7.35)	20.37**	.29	✔	✔	
Extraversion	45.59 (6.18)	49.17 (6.31)	52.10 (5.56)	5.79**	.10	✔		
Openness	48.79 (9.44)	50.18 (6.60)	52.10 (6.46)	.07	.00			
Agreeableness	49.29 (6.58)	50.58 (6.98)	52.16 (5.71)	.27	.01			
Conscientiousness	47.90 (7.55)	46.68 (7.90)	51.06 (5.45)	3.45*	.06		✔	

Negative life events (LES)^b^	*n* = 18	*n* = 30	*n* = 33					
Dependent	2.22 (1.34)	2.75 (1.53)	1.32 (1.17)	7.81**	.17		✔	
Independent	.50 (.49)	.33 (.48)	.35 (.41)	1.60	.04			

Coping skills (CISS)^c^	*n* = 21	*n* = 36	*n* = 47					
Task-oriented	45.83 (6.31)	47.57 (8.03)	51.60 (7.20)	2.81	.05	✔	✔	
Emotion-focused	53.72 (6.54)	51.88 (7.47)	46.19 (6.08)	6.71**	.12	✔	✔	
Avoidance-oriented	46.72 (4.99)	54.01 (8.48)	45.38 (7.60)	13.93**	.22		✔	✔

Social support (ASSI)^d^	*n* = 21	*n* = 37	*n* = 47					
Size of social network	9.17 (5.47)	10.73 (5.86)	17.20 (9.22)	6.20**	.11	✔	✔	
Satisfaction with social network	26.04 (2.90)	25.59 (2.92)	28.27 (1.85)	10.84**	.18	✔	✔	
Amount of social contact	5.87 (6.44)	7.64 (5.77)	12.44 (8.10)	4.41**	.08	✔	✔	
Satisfaction with social contact	4.10 (.80)	4.24 (.74)	4.70 (.47)	5.25**	.09	✔	✔	

Marital adjustment (DAS)^e^	*n* = 19	*n* = 26	*n* = 46					
Consensus	48.35 (7.07)	51.88 (6.16)	53.52 (5.44)	5.89**	.12	✔		✔
Affectional expression	7.78 (1.84)	8.31 (2.78)	9.23 (1.95)	3.84*	.08	✔		
Satisfaction	31.68 (8.08)	35.81 (7.25)	39.72 (4.22)	10.88**	.20	✔	✔	✔
Cohesion	13.14 (4.72)	14.69 (5.00)	16.15 (3.32)	3.50*	.07	✔		

Verbal aggression (CTS)^f^	*n* = 19	*n* = 26	*n* = 45					
Inter-partner	22.85 (19.60)	15.24 (19.12)	9.44 (9.34)	2.31	.05	✔		

Due to non-independence of data and to obtain a global estimate of couple functioning, mean scores across all available parents were used for these analyses when there was more than one parent in a family [number of single-parent families = 11 (7 BD)]

T-scores were used for the Personality and Coping Skills subcategories, *p < .05, ** p < .01

BD: bipolar disorder; PD: personality disorder; ✔: significant group difference

^a^From the NEO Personality Inventory-Revised

^b^From the Life Events Scale

^c^From the Coping Inventory of Stressful Situations

^d^From the Arizona Social Support Interview

^e^From the Dyadic Adjustment Scale

^f^From the Conflict Tactic Scale

#### Couples in which one partner presents BD and one MDD

As presented in Table 4, among the couples with one partner with BD, those that included a partner with MDD differed on only two measures from the couples without MDD: lower scores for agreeableness (6% of the variance) and less affection in the marital relationship (16% of the variance). These two findings are noteworthy. In the previous analyses comparing all couples with BD vs. controls (Table 2), group membership accounted for none or only 7% of the residual variance in agreeableness and marital affection, respectively. Generally, relative to healthy control couples, the couples with one partner with BD, with and without a partner with MDD, were similar.

**Table 4:** Comparisons of personality traits and psychosocial functioning of couples in which one partner presents BD and the other major depression, couples in which one partner presents BD and the other no major depression, and healthy control couples

	Couples					Couples with a partner with BD and MDD vs healthy control couples	Couples with a partner with BD no MDD vs healthy control couples	Couples with a partner with BD and MDD vs couples with a partner with BD no MDD
	One partner with BD		Two healthy control partners					
	MDD	No MDD						
	*M *(SD)	*M *(SD)	*M *(SD)	*F*	*η*^2^			
Personality (NEO-PI-R)^a^	*n* = 18	*n* = 40	*n* = 47					
Neuroticism	57.07 (7.13)	54.61 (7.53)	44.94 (7.35)	18.82**	.27	✔	✔	
Extraversion	48.02 (6.64)	47.81 (6.44)	52.10 (5.56)	3.87*	.07		✔	
Openness	49.49 (7.06)	49.76 (8.06)	52.10 (6.46)	.08	.00			
Agreeableness	47.34 (8.41)	51.35 (5.64)	52.16 (5.71)	3.09*	.06	✔		✔
Conscientiousness	48.00 (6.05)	46.7 (8.42)	51.06 (5.45)	3.30*	.06		✔	

Negative life events (LES)^b^	*n* = 16	*n* = 32	*n* = 33					
Dependent	2.25 (1.30)	2.70 (1.54)	1.32 (1.17)	7.19**	.16	✔	✔	
Independent	.38 (.50)	.41 (.48)	.35 (.41)	.33	.01			

Coping skills (CISS)^c^	*n* = 18	*n* = 39	*n* = 47					
Task-oriented	46.28 (6.21)	47.23 (7.99)	51.60 (7.20)	2.85	.05	✔		
Emotion-focused	51.98 (6.02)	52.83 (7.65)	46.19 (6.08)	6.74**	.12	✔	✔	
Avoidance-oriented	51.26 (7.21)	51.36 (8.64)	45.38 (7.60)	7.03**	.12	✔	✔	

Social support (ASSI)^d^	*n* = 18	*n* = 40	*n* = 47					
Size of social network	9.42 (5.42)	10.50 (5.89)	17.20 (9.22)	6.39**	.11	✔	✔	
Satisfaction with social network	26.22 (2.73)	25.54 (2.97)	28.27 (1.85)	10.98**	.18	✔	✔	
Amount of social contact	6.23 (4.92)	7.34 (6.49)	12.45 (8.10)	4.52**	.08	✔	✔	
Satisfaction with social contact	4.19 (.686)	4.19 (.80)	4.70 (.496)	5.12**	.09	✔	✔	

Marital adjustment (DAS)^e^	*n* = 15	*n* = 30	*n* = 47					
Consensus	49.97 (5.33)	50.60 (7.39)	53.52 (5.44)	3.27*	.07	✔	✔	
Affectional expression	6.72 (2.94)	8.77 (1.79)	9.23 (1.95)	8.34**	.16	✔		✔
Satisfaction	32.83 (6.65)	34.68 (8.35)	39.72 (4.22)	8.34**	.16	✔	✔	
Cohesion	13.21 (5.10)	14.45 (4.82)	16.15 (3.32)	3.06	.07	✔		

Verbal aggression (CTS)^f^	*n* = 15	*n* = 30	*n* = 45					
Inter-partner	20.95 (16.63)	17.28 (20.93)	9.44 (9.34)	2.02	.05			

Due to non-independence of data and to obtain a global estimate of couple functioning, mean scores across all available parents were used for these analyses when there was more than one parent in a family [number of single-parent families = 11 (7 BD)]

T-scores were used for the Personality and Coping Skills subcategories, *p < .05, **p < .01

BD: bipolar disorder; MDD: major depression disorder; ✔: significant group difference

^a^From the NEO Personality Inventory-Revised

^b^From the Life Events Scale

^c^From the Coping Inventory of Stressful Situations

^d^From the Arizona Social Support Interview

^e^From the Dyadic Adjustment Scale

^f^From the Conflict Tactic Scale

## Discussion

The present study compared parents having BD and their intimate partners with healthy control parents and their partners on a comprehensive battery of psychosocial measures important for adaptive family functioning, including mental disorders, personality traits, negative life events, coping skills, social support, marital adjustment, and verbal aggression. To assess family-wide risk, we conducted exploratory analyses examining the same variables within couples rather than individuals, comparing couples having a parent with BD with those having no mental disorders. Notably, comorbid personality disorders among the participants with BD and the presence of MDD in their partners were examined, in order to determine how these two factors, in conjunction with increased heritability (Merikangas et al. 1988), might impact the family environment.

In line with previous research (Bender et al. 2010; Fletcher et al. 2013; MacQueen et al. 2001), parents with BD displayed more maladaptive personality traits, more dependent stressful life events, ineffective coping strategies, smaller social networks and fewer social contacts that provided lower levels of satisfaction than healthy control parents. In addition, they reported that their marital relationships were unsatisfactory and that they were verbally abused by their partners. Thus, even between acute episodes of BD, these parents seem to elicit high levels of stress. Moreover, they demonstrate ineffective coping strategies and receive little social support from those around them, including their intimate partners.

As a unique focus of the present study, the intimate partners of the parents with BD differed in multiple ways from the partners of healthy control parents. Consistent with the literature on assortative mating (Butterworth and Rodgers 2008; Mathews and Reus 2001; Nordsletten et al. 2016), 56% of the partners of parents with BD presented current or past axis I disorders, and 10% presented axis II disorders. Additionally, they presented high levels of neuroticism, low levels of extraversion, and frequent use of emotion-focused coping, relative to healthy control partners. The intimate partners of the parents with BD also had few contacts outside the couple, and thereby little social support, while acknowledging that their marital relationship was unsatisfactory and characterized by high levels of verbal abuse. Marital problems, high levels of emotion-focused coping and failure to establish social support networks by the partners of adults with BD may result from a heightened perception of the burden imposed by their partners (Borowiecka-Karpiuk et al. 2014; Perlick et al. 2007, 2016; Reinares et al. 2006). Alternatively, or additionally, the maladaptive behaviors of the partners, like those of adults with BD, may be associated with high levels of neuroticism. For example, individuals high in neurotic traits tend to respond to stress with negative affect (Jacobs et al. 2011) that in turn is linked to emotion-focused coping strategies. Similarly, neuroticism is associated with depression symptoms that are in turn linked to low levels of social support (Stice et al. 2004).

The portrait of the intimate partners of parents with BD indicates that they confer risk for mental disorders, neuroticism, and low psychosocial functioning in the OBD, rather than providing a buffering effect. The presence of heritable mental disorders and neuroticism among the intimate partners of parents with BD suggest that they also may transmit genes to the OBD that increase their liability for similar disorders and the trait of neuroticism (Kieseppä et al. 2004; Song et al. 2015). Importantly, the presence of high neuroticism in both parents has been associated with negative outcomes in their offspring (Ellenbogen et al. 2010). Indeed, parents’ neuroticism is associated prospectively with poor interpersonal functioning and higher rates of risky sexual behaviours in late adolescence and early adulthood among their offspring (Ostiguy et al. 2012; Nijjar et al. 2016). Further, OBD show greater sensitivity to their parents’ emotionality than offspring of healthy control parents (Ostiguy et al. 2012). Moreover, the intimate partners of parents with BD, but not the parents with BD, reported decreased extraversion compared to healthy controls. This finding is important as low extraversion is associated with negative health outcomes, anxiety disorders, and depressive symptoms, all of which might exacerbate an already stressful home environment (Bienvenu et al. 2007; Hakulinen et al. 2015).

Taken together, the present findings indicate that both parents model maladaptive behaviors such as emotion-focused coping skills, and experience low social support and marital difficulties. Therefore, these findings suggest that parents with BD *and* their intimate partners may benefit from individual treatments aimed at developing effective coping skills, social support networks, and a satisfying marital relationship. These findings are consistent with and further support the use of efficacious adjunct interventions for BD targeting families, such as Family-Focused Therapy for BD, rather than pharmacotherapy alone (Miklowitz et al. 2003, 2017). Interventions targeting neuroticism in both parents, although more difficult, might be particularly pertinent given its robust associations with environmental outcomes (Tang et al. 2009).

Contrasting individual parent reports of marital adjustment in the dyad revealed interesting differences in perspective. Similar to previous research (Lam et al. 2005; Whisman 2007), both parents with BD and their intimate partners reported low marital satisfaction relative to healthy controls. However, differences in partner reports surfaced with regards to other aspects of marital functioning. Parents with BD, but not their intimate partners, qualified their relationships as having infrequent expressions of affection and sexual desire and few common activities and interests within the couple, relative to healthy controls. This result concurs with previous studies reporting heightened difficulties adjusting to the shifts in sexual arousal/desire during manic and depressive episodes of the affected partner, as reported by their intimate partners (Lam et al. 2005). Consistent with previous literature (Perlick et al. 2016), intimate partners of parents with BD often experience depressive symptoms themselves, which includes decreased interest for activities and sex. Therefore, this may be another way by which the partner with BD may be unsatisfied in these areas of their relationship. From the perspective of the intimate partner of adults with BD, difficulties in marital adjustment are driven by disagreements on what is important in their relationship. This may be a result of the burden of caregiving often experienced by intimate partners of patients with BD, including increased responsibilities in maintaining finances, household routines, and childcare (Perlick et al. 2007).

Surprisingly, the presence of comorbid PDs in adults with BD, or MDD in their partner, altered few of the differences between couples with BD and healthy control couples. Specifically, inter-partner verbal abuse was greatest among couples with BD and PDs, which is consistent with studies showing high rates of personality disorder among domestic violence perpetrators (Gibbons et al. 2011). Marital difficulties in couples with BD and comorbid PD or a partner with MDD were greater than couples with BD only. These findings suggest that the marital adjustment and verbal abuse associated with BD may be driven, in part, by mental disorders other than BD within these couples. With respect to PD, this is not surprising as symptoms of PDs in an intimate partner within community samples have been associated with marital dysfunction and low satisfaction (South et al. 2008; Whisman and Schonbrun 2009).

Although this is, to the best of our knowledge, the first comprehensive study of parents having BD and their intimate partners, there are a number of study limitations. The results of the study can only be generalized to middle-class and Caucasian parents with BD and their partners who care for children. These individuals may differ from other adults with BD who have no children, or families living in poverty or from minority communities. For example, the participants with BD in the current study showed lower rates of anxiety disorders than previously reported among adults with BD (McElroy et al. 2001). Another limitation is the relatively small sample size that may have prevented the detection of effects when taking account of mental disorders other than BD in couples. Therefore, readers should mainly focus on effect sizes when interpreting these results and interpret small effects sizes with caution. Finally, except for the assessment of mental health and personality disorders, the study relies solely of self-report measures. Observational methods or ratings from other sources would strengthen the reported findings.

## Conclusion

In conclusion, adults with BD appear to select intimate partners with similar maladaptive personality traits, ineffective coping skills and who demonstrate high levels of verbal aggression. Moreover, both adults with BD and their intimate partners report a paucity of social support. These findings indicate that the intimate partners, as well as the partners with BD, could *both* benefit from individual treatments aimed at lowering emotionality and verbal aggression, increasing social support, and improving effective coping skills. Not surprisingly, both partners in these couples report difficulties and low levels of satisfaction in their relationships, which was further exacerbated (along with verbal aggression) by comorbid PDs and or a having a partner with MDD. Thus, the present findings indicate that couple and family therapy are also warranted. Many of the suggested intervention might improve family functioning and by extension the health of their children. Indeed, strong social support systems in parents with BD during middle childhood were found to act as a protective factor against the future development of substance use symptoms in OBD in early adulthood (Trespalacios et al. manuscript in preparation).

## Data Availability

The datasets generated and/or analyzed during the present study are not publicly available.
